# Long-term outcomes of metacarpal fractures surgically treated using bioabsorbable plates: a retrospective study

**DOI:** 10.1186/s12891-020-03841-x

**Published:** 2020-12-07

**Authors:** Kenji Kosugi, Yukichi Zenke, Takafumi Tajima, Yoshiaki Yamanaka, Kunitaka Menuki, Akinori Sakai

**Affiliations:** Department of Orthopaedic Surgery, University of Occupational and Environmental Health, 1-1 Iseigaoka, Yahatanishi-ku, Kitakyushu, 807-8555 Japan

**Keywords:** Bioabsorbable plates, Metacarpal fractures, Long-term outcomes, U-HA/PLLA

## Abstract

**Background:**

Implants made from bioabsorbable unsintered hydroxyapatite and poly-L-lactate composites (u-HA/PLLA) are widely used in the oral, maxillofacial, and orthopedic fields. This study assess the long-term (> 5 years) outcomes of patients with metacarpal fractures who were surgically treated using bioabsorbable plates and screws (Super-Fixsorb MX40 mesh; Teijin Medical Technology, Osaka, Japan).

**Methods:**

A retrospective analysis of six patients with eight metacarpal fractures treated with bioabsorbable plates was done. All patients were followed for more than 5 years post-surgery. The clinical outcomes were evaluated using Q-DASH scores and the grip strength (GS): opposite side ratio. The resorption status of implants was assessed on plain computed tomography (CT) scans at final follow-up appointments.

**Results:**

The mean age of the patients at the time of surgery was 29.5 years (16–54), and the median follow-up period was 81.8 months (68–101). All fractures united without displacement after an average of 3.5 months, and there were no implant specific complications associated with the use of absorbable plates. The mean grip strength ratio was 85.1% (56.8–104.5). The mean Q-DASH scores of 11.36 points (0–34.09) was good in all but two patients. We also observed that it took more than 8 years for the plates to be absorbed completely.

**Conclusions:**

This study demonstrates that the process of bioabsorption in metacarpal fractures might be completed in about 8 years, and the absorption speeds were different inside and outside of the bone. The bioabsorbable plates are more cost-effective than metallic implants. The potential for bioabsorbable plates to be used in various clinical procedures is promising.

**Supplementary Information:**

The online version contains supplementary material available at 10.1186/s12891-020-03841-x.

## Background

Metacarpal fractures are common, and most of them can be treated successfully without surgery [1]. However, open reduction and internal fixation is a common surgical treatment for unstable metacarpal fractures, and the use of metallic implants is considered the gold standard for operative treatment [2, 3]. However, metallic implants may interfere with extensor tendon gliding and induce metallosis and allergic reactions [4]. Surgical removal of the implant is frequently required to avoid complications; moreover, titanium plates and screws are often difficult to remove after extended periods [5, 6].

Unsintered hydroxyapatite and poly-L-lactate composites (u-HA/PLLA) are resorbable osteosynthetic bone fixation implants with osteoconductive properties that promote replacement with new bone [7]. u-HA/PLLA constructs are radiopaque and have biomechanical strength equal to or greater than that of titanium plates [8]. Therefore, u-HA/PLLA composites are widely used in oral and maxillofacial, and orthopedic fields, especially fractures of the upper extremities such as metacarpal fractures and distal radial intra-articular fractures [8–11]; however, in the treatment of unstable metacarpal fractures, most surgeons still use metal plates. Animal studies have shown that composite u-HA/PLLA rods can be completely replaced by new bone in the distal femoral condyle of rabbits within 7.3 years without producing adverse reactions [7]. Human studies in oral and maxillofacial fields have shown that surgically implanted u-HA/PLLA composites were absorbed in 6 years [12]. However, reports have described late-onset, foreign body reactions such as infections and aseptic swelling [13–15]. Regarding orthopedics, no previous report has detailed long-term clinical outcomes using u-HA/PLLA plates.

This study aims to evaluate the long-term (> 5 years) outcomes of surgically treated metacarpal fractures using bioabsorbable plates.

## Methods

From September 2009 to January 2013, total 32 patients with unstable metacarpal fractures were treated at the study hospital using bioabsorbable plates (Super-Fixsorb MX40 mesh; Teijin Medical Technology, Osaka, Japan). Twenty-six patients finally obtained bone union and full of range of motion without pain, therefore, completed follow-up within 1 year. Six patients (four males and two females, eight fingers), with follow-up for more than 5 years, were included in this study.

### Surgical procedures

All patients underwent similar surgeries. Patients were either administered general or regional anesthesia using Ropivacaine, and a curved, longitudinal skin incision was made on the dorsal part of the hand. Open reductions were performed, and fractures were temporarily fixed using Kirschner wires that were 1.0 mm in diameter. Bioabsorbable mesh sheets (50 mm × 50 mm × 0.7 mm, height × width × depth) were cut into the appropriate shape for fixation of the fracture, and the sheets were bent using a hot water bath (68 °C) to make a one-third or semi-tubular plate. After placing the sheets in a hot water bath for about 15 min, they were designed to anatomically fit the curved surface of the fractured metacarpal. Plates were anatomically fitted to curved surfaces of metacarpal bones (Additional File 1), and each was fixed with either four or six bioabsorbable, 2.0-mm diameter cortical screws (Super-Fixsorb MX30; Teijin Medical Technology, Osaka, Japan). Each plate was covered with fascia from the interosseous muscle to avoid irritation of the extensor tendon.

Postoperatively, the wrist and metacarpophalangeal joints were immobilized with a splint until swelling and pain had sufficiently diminished, which typically took about 1 week. Active range of motion exercises were then initiated to the extent that the patients could tolerate.

Clinical outcomes were evaluated through the assessment of the active range of motion (ROM) of the wrist and forearm, grip strength ratio (percentage of the unaffected side), and Quick-Disabilities of the Arm, Shoulder, and Hand (Q-DASH) score [16]. Those who cannot grip completely were defined as having a limited ROM. We judged Q-DASH score of 0 points as good. Patients were evaluated at their final follow-up appointment. Bone union on plain radiograph and complications such as infection and persistent swelling were assessed on follow-up visits. Resorption status of plates and screws were also assessed at final follow-up visits on plain computed tomography (CT) scans. We defined that the plate was completely absorbed when the shape of the plate could not be traced by 3D-CT. The data were expressed with average and standard deviation or median and minimal-maximal value.

## Results

The clinical results for all patients are summarized in Table 1. The mean age of the patients at the time of surgery was 29.5 years (range, 16–54 years), and the mean follow-up period was 81.8 months (range, 68–101 months). All fractures were united without displacement after an average of 3.5 months, and there were no implant specific complications such as inflammation and persistent swelling reported. The mean grip strength ratio (%) was 85.1 ± 17.5(range, 56.8–104.5). The mean Q-DASH scores (points) was 11.36 (range, 0–34.09). The score was good for all but two cases. In cases in which these were poor, patients had limited ranges of motion and poor recovery of grip strength ratios. On studying persistence or resorption it was found that both plates and screws typically persist for more than 6 years post-surgery. In fact, remnants of plate were observable 7 years post-surgery. The plate was completely absorbed 8 years and 5 months post-surgery, however, only a small quantity of screws remained within the bone.

**Table 1:** Assessment of clinical outcomes of all patients recorded during the final follow-up appointment

Case	Sex	Age	Affected hand	Affected finger	Dominant hand	Bone union	F/u	Limited ROM	G-S ratio	Q-DASH
1	M	16	Right	Little	Right	2 M	5Y8 M	No	79.2	0
2	F	54	Right	Ring	Right	3 M	6Y5M	No	104.5	0
3	M	17	Right	Ring	Right	2 M	6Y7M	No	100.7	0
4	M	18	Left	Ring	Right	3 M	6Y8 M	Yes	78.5	34.09
5	F	52	Right	Little	Right	3 M	7Y2 M	No	91.1	0
6	M	20	Right	Middle Ring Little	Right	8 M	8Y5M	No	56.8	34.09
Ave.		29.5 ± 18.2				3.5 M	6Y9M		85.1 ± 17.5	11.36 ± 17.60

Values are presented as means ± SD

*Abbreviations*: *Ave.* Average, *M* Male, *F* Female, *F/u* Follow-up, *ROM* range of motion, *G-S* grip strength, *Q-DASH* Quick Disabilities of the Arm, Shoulder, and Hand

### Case presentation

#### Case 1

**Fig. 1:**
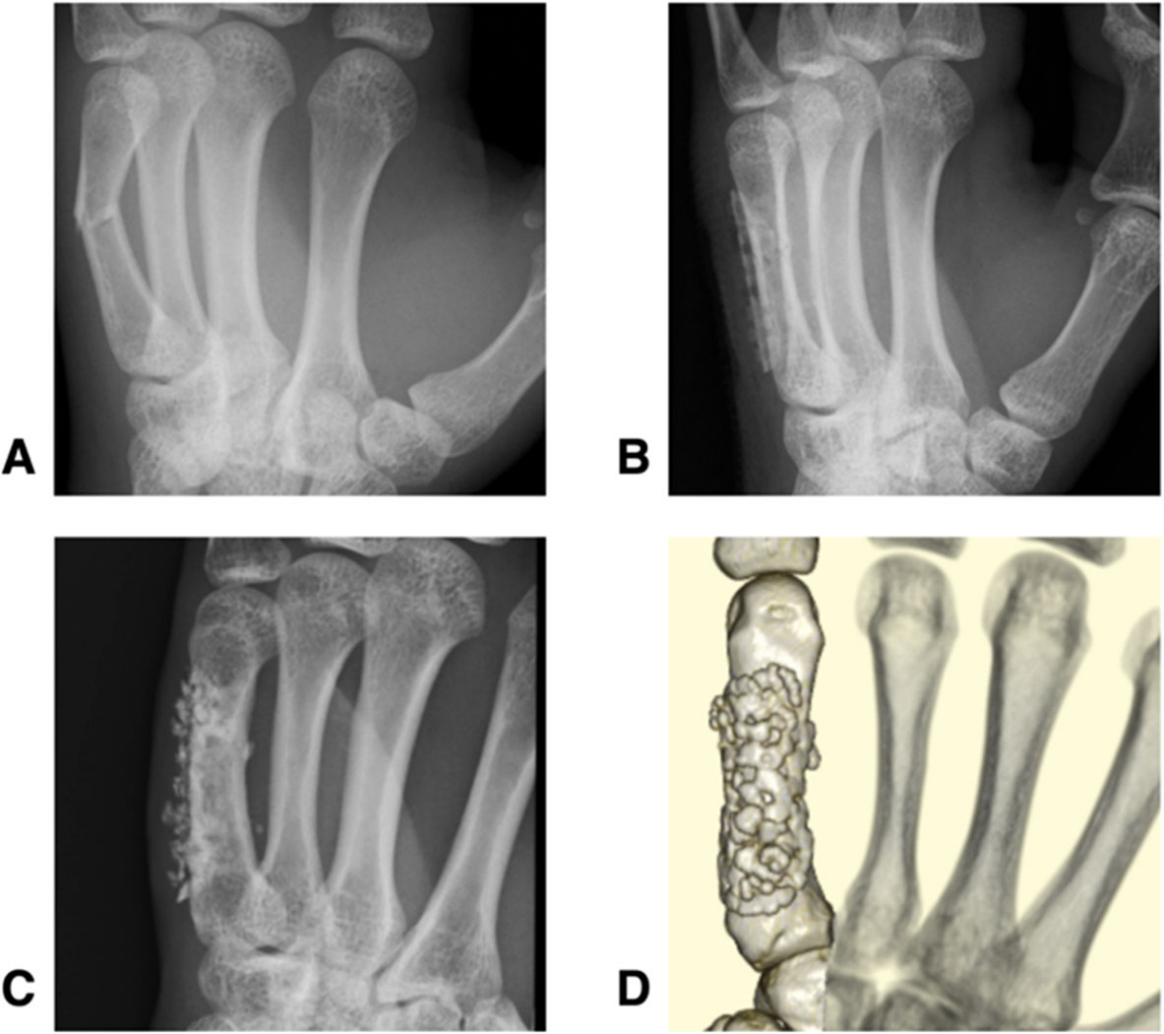
Radiographic images of a repaired transverse metacarpal fracture of the left little finger in a 16-year-old male (Case 1). The preoperative radiograph (**a**), postoperative radiograph (**b**), and final follow-up radiograph (**c**), which was taken 5 years 8 months post-surgery are pictured. A final follow-up 3D-computed tomography image (**d**) is also shown

A 16-year-old male presented with a transverse metacarpal fracture of the left little finger as a result of bruising while playing baseball. We treated it with a bioabsorbable plate made from a single mesh sheet. The plate was anatomically fitted to the curved surface of the fractured bone. At final follow-up visit, 5-years and 8-months post-surgery, the active ROM of his wrist and forearm was full, and the grip strength ratio was 79.2%. The Q-DASH score was 0 points. CT imaging revealed that both the plate and screws clearly remained (Fig. 1).

#### Case 2

A 54-year-old woman had an oblique metacarpal fracture of the left ring finger as a result of a fall from standing height. Operation was performed as described in Case 1. At final follow-up visit, 6-years and 5-months post-surgery, the active ROM was full, and the grip strength ratio was 104.5%. The Q-DASH score was 0 points. Both the plate and the screws were clearly visible in plain CT images.

#### Case 3

**Fig. 2:**
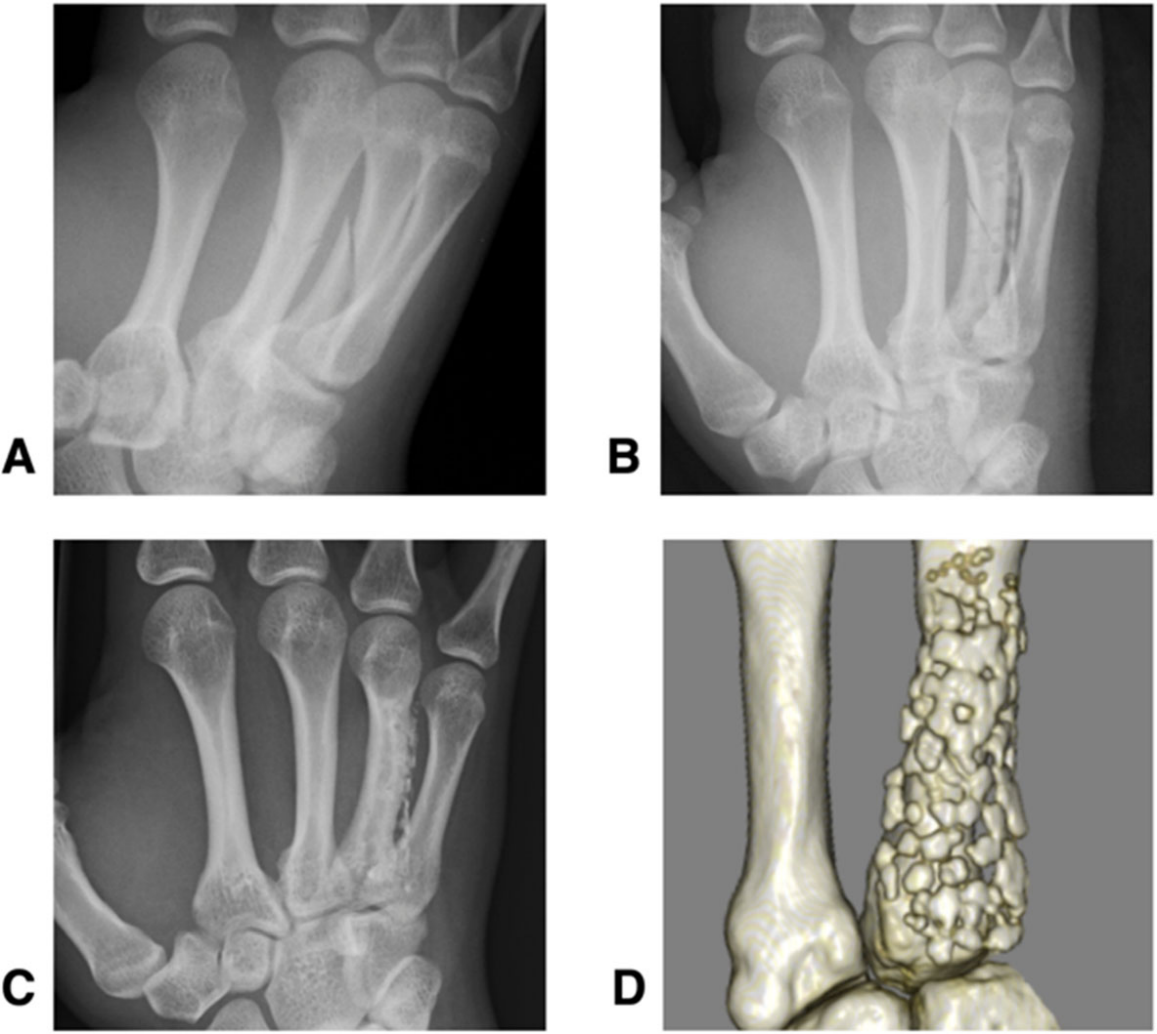
Radiographic images of a 17-year-old male who had an oblique metacarpal fracture of the right ring finger (Case 3). The preoperative radiograph (**a**), postoperative radiograph (**b**), final follow-up radiograph (**c**) (taken 6 years 7 months post-surgery) and a final follow-up 3D-computed tomography image (**d**) are shown

A 17-year-old man had an oblique metacarpal fracture of the right ring finger as a result of sliding head-first while playing baseball. Operation was performed. At final follow-up visit, 6-years and 7-months post-surgery, the active ROM was full, and the grip strength ratio was 100.7%. The Q-DASH score was 0 points. Plain CT revealed that most of the implanted plate and screws remained (Fig. 2).

#### Case 4

An 18-year-old man had an oblique metacarpal fracture of the left ring finger as a result of a diving catch while playing baseball. Operation was performed. At final follow-up visit, 6-years and 8-months post-surgery, active flexion ROM of the MCP joint was 80 degrees, therefore he could not grip completely. There were no physical findings that suggested adhesion of the extensor tendon, and there were no abnormalities observed, such as screw protrusion. The grip strength ratio was 78.5%, and the Q-DASH score was 34.09 points. On plain CT images, both the plate and the screws were visible.

#### Case 5

**Fig. 3:**
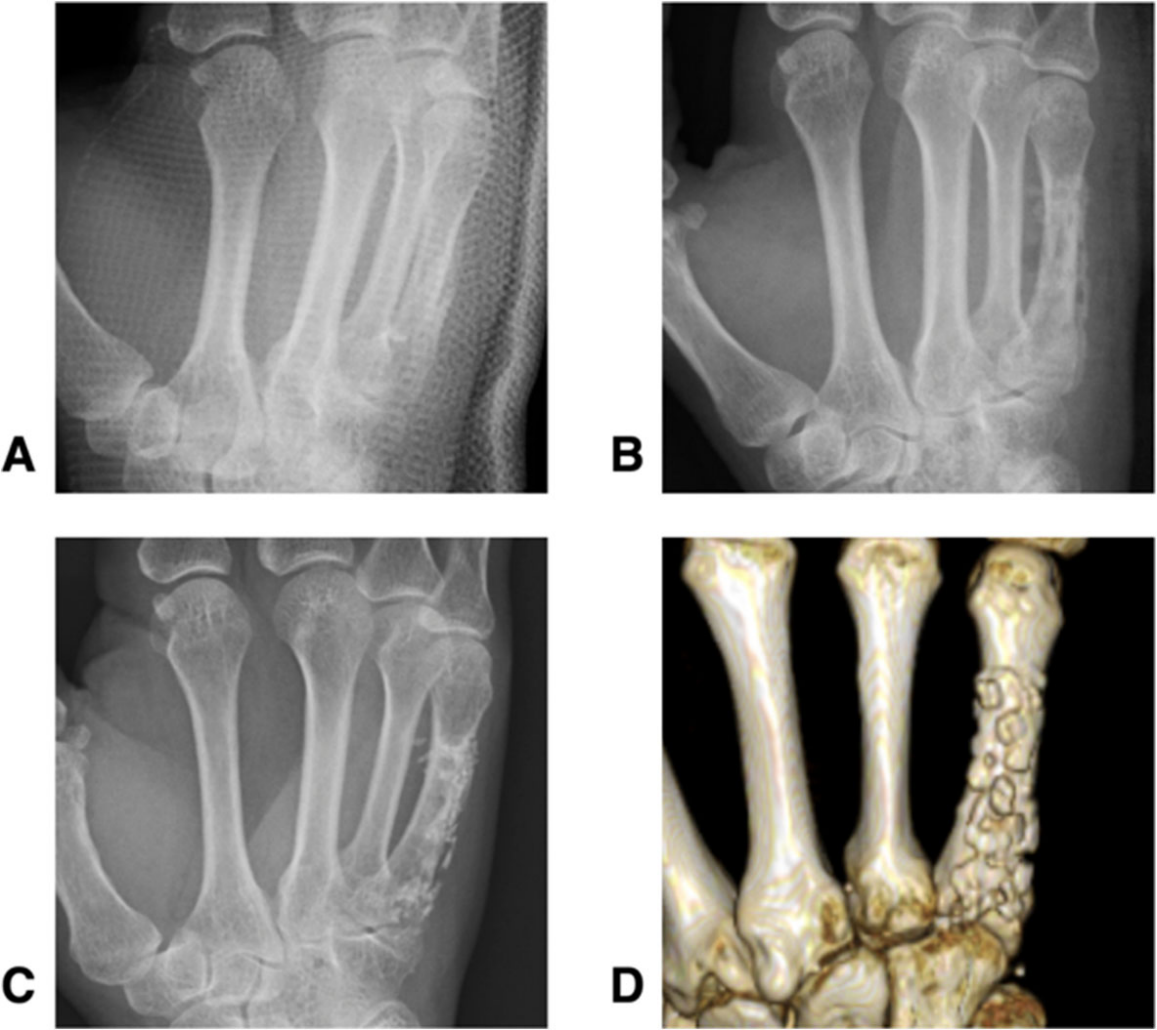
Radiographic images of a 52-year-old female with an oblique metacarpal fracture of the right little finger (Case 5). The **a** preoperative radiograph, **b** postoperative radiograph, **c** final follow-up radiograph (Post 7 years 2 months), and **d** final follow-up 3D-computed tomography images are shown

A 52-year-old woman had an oblique metacarpal fracture of the right little finger as a result of a bruise while playing volleyball. Operation was performed. At final follow-up visit, 7-years and 2-months post-surgery, the active ROM was full, the grip strength ratio was 91.1%, and the Q-DASH score was 0 points. On plain CT, the plate and the screws were only slightly visible (Fig. 3).

#### Case 6

**Fig. 4:**
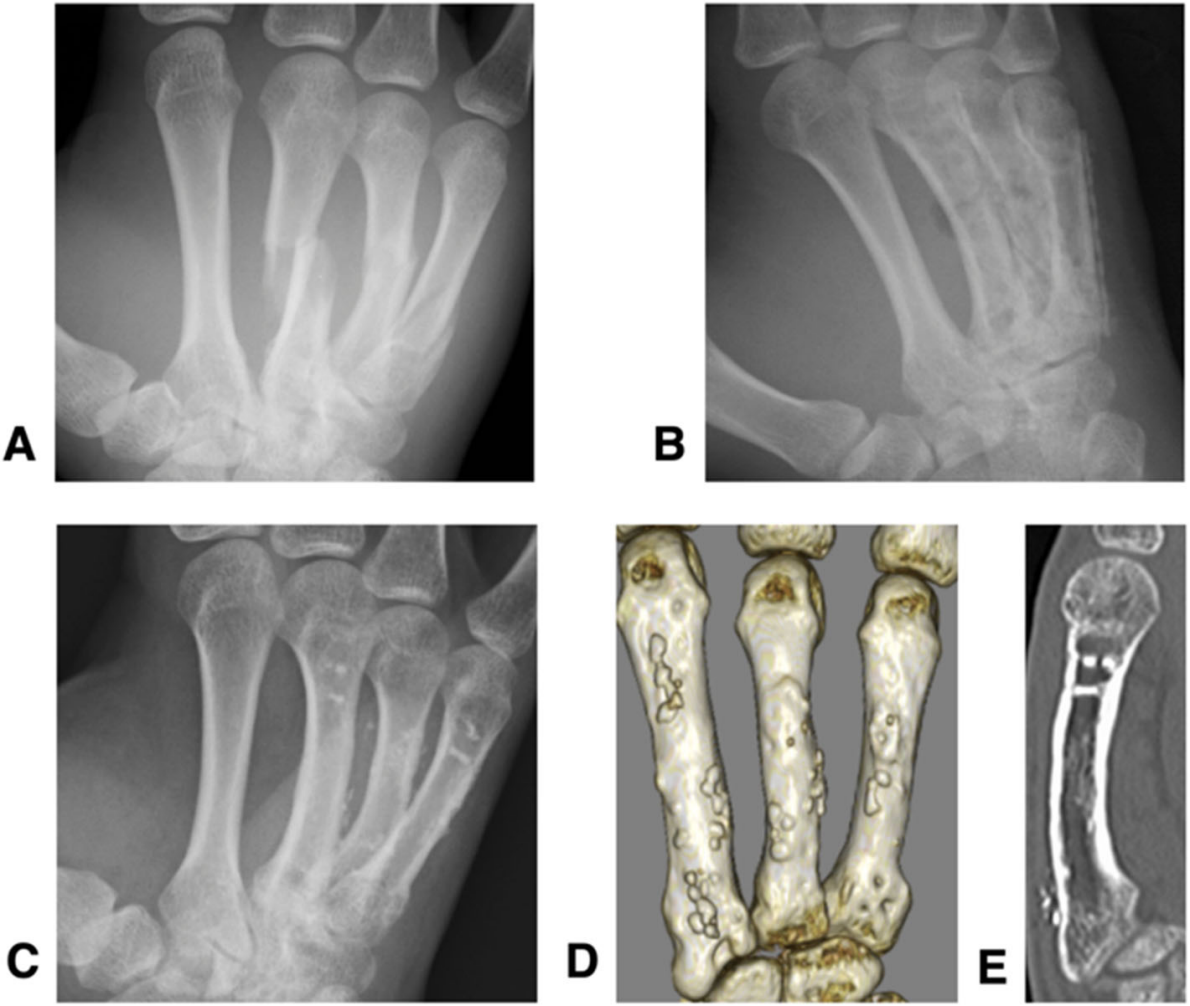
Radiographic images from a 20-year-old male with a comminuted metacarpal fracture of the left middle finger and oblique metacarpal fractures of the left ring and little fingers (Case 6). The **a** preoperative radiograph, **b** postoperative radiograph, **c** final follow-up radiograph (taken 7 years 2 months post-surgery), **d** final follow-up 3D-computed tomography and **e** final follow-up plain CT images are shown

A 20-year-old man had a comminuted metacarpal fracture of the left middle finger and oblique metacarpal fractures of the left ring and little fingers as a result of an accident that occurred at work. Operations for all injured fingers were performed. At final follow-up visit, 8-years and 5-months post-surgery, the active ROM was full; however, the grip strength ratio was 56.8%, and the Q-DASH score was 34.09 points. Plain CT clearly revealed that the plates had been completely absorbed, and only screws remained slightly visible within the bone (Fig. 4).

## Discussion

u-HA/PLLA composites are mainly used to treat fractures of the upper extremities [8, 17]. Unstable metacarpal fractures are commonly treated by open reduction and internal fixation using rigid devices, such as titanium plates [2, 3]. However, these implants are often associated with high complication rates [5, 6]. u-HA/PLLA bioabsorbable implants have been introduced as a reliable alternative to titanium plates. These absorbable implants are designed to provide several clinical advantages compared to metallic implants [8]. Firstly, mesh sheets made from u-HA/PLLA are very thin (0.7 mm thickness); therefore, the associated risk of soft tissue irritation is lower than that of titanium plates. Secondly, mesh sheets are malleable and can be placed in the best possible position to cover a fracture according to the fracture pattern. Furthermore, there are many screw holes in the mesh sheets. Therefore, surgeons have more flexibility for screw placement. The u-HA/PLLA material also serves as a good alternative for patients allergic to metal. Above all, there is no need to remove the u-HA/PLLA bioabsorbable plates.

It has already been demonstrated that u-HA/PLLA bioabsorbable plate constructs have sufficient mechanical strength to stabilize metacarpal fractures [8]. The bending strength and stiffness of semi-tubular and one-third tubular bioabsorbable plate constructs were comparable to those of titanium plates. Further, the strengths of plates were shown to remain comparable to that of cortical bone for 6 months [11], which is more than the 3–4 months required for bone healing. In fact, good clinical results using bioabsorbable plates to repair metacarpal fractures have been reported [8]. In these studies, bioabsorbable plates were used to repair diaphyseal and proximal fractures. Fractures near the joints should be handled with care, because bioabsorbable plates have no locking system, and there is a large amount of cancellous bone in the proximal metacarpal bone near joints. Therefore, there is a possibility that screws may fall out or loosen. In addition, tapping must be performed to fix screws, and care must be taken to avoid tightening screws too strongly because the shear strength of bioabsorbable screws is weaker than that of titanium screws, and they may twist as a result of breakage between the screw shaft and head. In these cases, a rescue technique may be used to cauterize the screw stump using an electrosurgical knife to integrate it within the bioabsorbable plate. In the past, breakage of screw heads occurred in a small number of our patients; however, this complication has not recently occurred.

Delayed foreign body reactions such as inflammation and persistent swelling have been reported occasionally in the oral and maxillofacial surgical fields [13]. These are thought to be caused by mechanical irritation due to protrusion of screws that had not been degraded, and inflammatory reactions that occur throughout the process of degradation and absorption. In orthopedic literature, one study with mean follow-up period of 45.7 months (range, 34–61 months) showed that foreign body reactions did occur in four of nine patients that required second procedures to remove implants [18]. In this study, no implant-specific complications were reported, which indicates that operation site likely affects surgical outcomes. In oral and maxillofacial surgery, the presence of oral bacteria cannot be ignored [19]. In fact, the surgical site infection frequency of hand fractures was reported to be 1% [20], whereas that of mandibular closed fractures was reported to be 1.4–33.4% [21, 22]. The surgery was performed as described to prevent mechanical irritation, however, other factors were unknown.

In this study, two cases yielded low Q-DASH scores. One case had limited range of motion. There were no physical findings that suggested adhesion of the extensor tendon, and there were no abnormalities observed, such as screw protrusion. Postoperative therapy was more likely to affect the observed outcome than technical problems. In another case, poor recovery of grip strength ratio was observed; however, the patient had multiple fractures which could have led to poor level of recovery.

In this study, in the case of metacarpal fractures, we found that the process of bioabsorption might be completed in about 8 years. Previous studies conducted on wrist arthritis using four-corner fusion for scapholunate advanced collapse and scaphoid nonunion advanced collapse using bioabsorbable plates indicate that it took significantly fewer years (approximately 5 years) for absorption to take place after surgery [23]. These results suggest that the speed of absorption depends on the location of surgical intervention. We speculate that the less cancellous nature of metacarpal bones may be a factor that contributes to the differences observed between metacarpal and four-corner fusion absorption rates. However, there are no studies that have demonstrated that absorption speeds of implants within the bone differ from those placed outside the bone. This study suggests that cancellous bone volume may contribute to differences in plate bioabsorption rates.

This study had three limitations. Firstly, this study assessed a case series retrospectively, and the sample size was limited to six patients. Therefore, more cases should be included in future studies. Secondly, as mentioned above, no true locking system exists, and there is a possibility that screws may have fall out or loosen. In this study, there were no cases where screws backed out. Thirdly, control group was not included in this study. In spite of these limitations, one of the strengths of this study is long-term follow-up and the results of this study suggest that the use of bioabsorbable plates is an useful option for the treatment of displaced metacarpal fractures.

## Conclusion

This study demonstrates that the treatment of metacarpal fractures using bioabsorbable plates produces good outcomes, and in the case of metacarpal fractures, the process of bioabsorption might be completed in about 8 years. Bioabsorbable plates are custom-made implants and have several clinical advantages compared to metallic implants. Additionally, there is no need to remove the bioabsorbable plates, and it is possible to craft multiple original plates using only one mesh sheet; therefore, bioabsorbable plates are more cost-effective than metallic implants. The potential for bioabsorbable plates to be used in various clinical procedures is promising.

## Supplementary Information

**Additional file 1.** Crafting bioabsorbable plates for fixation of metacarpal fractures.

## Data Availability

The datasets used and/or analyzed during the current study are available from the corresponding author on reasonable request.

## Abbreviations

u-HA/PLLA: Unsintered hydroxyapatite and poly-L-lactate composites
Q-DASH: Quick-Disabilities of the Arm, Shoulder, and Hand
CT: Computed tomography
